# Strong impact of MammaPrint and BluePrint on treatment decisions in luminal early breast cancer: results of the WSG-PRIMe study

**DOI:** 10.1007/s10549-018-05075-x

**Published:** 2019-02-22

**Authors:** R. Wuerstlein, R. Kates, O. Gluz, E. M. Grischke, C. Schem, M. Thill, S. Hasmueller, A. Köhler, B. Otremba, F. Griesinger, C. Schindlbeck, A. Trojan, F. Otto, M. Knauer, R. Pusch, N. Harbeck, WSG-PRIMe investigators in Germany, Austria, Switzerland, E.-M. Grischke, N. Harbeck, C. Schem, O. Gluz, M. Thill, S. Hasmüller, A. Köhler, B. Otremba, F. Griesinger, C. Schindlbeck, T. Reimer, J. Krauter, O. Tomé, K. Friedrichs, U.-S. Albert, G. Gebauer, S. Ackermann, I. Scheffen, G. Kaltenecker, F. Overkamp, I. Schrader, J. Potenberg, H.-M. Enzinger, A. Trojan, F. Otto, R. Pusch

**Affiliations:** 1 Department of Gynecology and Obstetrics, Breast Center, University of Munich (LMU), CCC Munich, Munich, Germany; 2 West German Study Group GmbH, Moenchengladbach, Germany; 3 Brustzentrum Niederrhein, Evangelisches Krankenhaus Bethesda, Mönchengladbach, Germany; 4 Universitätsfrauenklinik Tuebingen, Tuebingen, Germany; 5 Universitätsklinikum Kiel, Frauenklinik, Kiel, Germany; 6 Agaplesion Markus Hospital, Frankfurt, Germany; 7 Ebersberg Clinic, Ebersberg, Germany; 8 Gemeinschaftspraxis für Hämatologie und Onkologie, Langen, Germany; 9 Onkologische Praxis Oldenburg, Oldenburg, Germany; 10 Klinikzentrum für Hämatologie und Onkologie, Oldenburg, Germany; 11 Klinikum Traunstein, Frauenklinik, Traunstein, Germany; 12 Brust-Zentrum Zürich, Zurich, Switzerland; 13 Tumor-und Brustzentrum ZeTuP and Brustzentrum Stephanshorn, St. Gallen, Switzerland; 14 Breast Center Kantonsspital St. Gallen, St. Gallen, Switzerland; 15 Ordensklinikum Linz, Linz, Austria; 16 University Hospital Cologne, Cologne, Germany

**Keywords:** Breast cancer, Diagnostic test, MammaPrint, BluePrint, Molecular profiling, Decision impact

## Abstract

**Purpose:**

The WSG-PRIMe Study prospectively evaluated the impact of the 70-gene signature MammaPrint® (MP) and the 80-gene molecular subtyping assay BluePrint® on clinical therapy decisions in luminal early breast cancer.

**Methods:**

452 hormone receptor (HR)-positive and HER2-negative patients were recruited (N0, N1). Physicians provided initial therapy recommendations based on clinicopathological factors. After prospective risk classification by MammaPrint/BluePrint was revealed, post-test treatment recommendations and actual treatment were recorded. Decisional Conflict and anxiety were measured by questionnaires.

**Results:**

Post-test switch (in chemotherapy (CT) recommendation) occurred in 29.1% of cases. Overall, physician adherence to MP risk assessment was 92.3% for low-risk and 94.3% for high-risk MP scores. Adherence was remarkably high in “discordant” groups: 74.7% of physicians initially recommending CT switched to CT omission following low-risk MP scores; conversely, 88.9% of physicians initially recommending CT omission switched to CT recommendations following high-risk MP scores. Most patients (99.2%) recommended to forgo CT post-test and 21.3% of patients with post-test CT recommendations did not undergo CT; among MP low-risk patients with pre-test and post-test CT recommendations, 40% did not actually undergo CT. Luminal subtype assessment by BluePrint was discordant with IHC assessment in 34% of patients. Patients’ State Anxiety scores improved significantly overall, particularly in MP low-risk patients. Trait Anxiety scores increased slightly in MP high risk and decreased slightly in MP low-risk patients.

**Conclusions:**

MammaPrint and BluePrint test results strongly impacted physicians’ therapy decisions in luminal EBC with up to three involved lymph nodes. The high adherence to genetically determined risk assessment represents a key prerequisite for achieving a personalized cost-effective approach to disease management of early breast cancer.

## Introduction

In the last decade, the use of genomic tests to determine the risk of recurrence in early-stage breast cancer has increased substantially [1]. Gene expression signatures categorize breast cancers into subtypes with increased biological homogeneity, supporting a more personalized approach [1, 2] to disease management than conventional clinicopathological factors [3].

MammaPrint (Agendia NV, the Netherlands) examines the expression levels of 70 genes [4, 5] to assess the risk of distant metastasis in breast cancer and classifies tumors as high vs. low risk. The BluePrint test (80-gene signature) performs a molecular sub-classification (Luminal, HER2, or Basal type) and (together with MammaPrint) identifies sub-populations with potentially distinct treatment response [6–8].

In the adjuvant setting, the quality of prognostic classification by MammaPrint has been evaluated in several retrospective and prospective studies [9–14]. In the neoadjuvant setting, the potential-added value of MammaPrint and BluePrint was shown in the Neoadjuvant Breast Registry Symphony Trial (NBRST), where intrinsic subtype was reclassified in 22% of tumors.

The prospective, randomized, international MINDACT trial (Microarray in Node Negative and 1 to 3 Positive Lymph Node Disease May Avoid Chemotherapy (EORTC 10041/BIG 3–04)) randomized patients with discordant risk classification (by clinical vs. MammaPrint risk assessment) into groups treated according to one or the other classifications. In particular, 5-year distant metastasis-free survival (5y-DMFS) was 95% in the clinically high-risk group randomized to receive no chemotherapy due to low-risk MammaPrint classification—thus achieving the primary endpoint (lower confidence limit > 92%) [15].

The MammaPrint and BluePrint tests are recommended by national and international guidelines to support adjuvant therapy decisions and are currently used in daily practice. In view of their potential for higher-quality chemotherapy decisions, MammaPrint and BluePrint use could have substantial impact on clinical practice. However, the impact of any testing strategy depends on adherence of physicians (and patients) to test results, particularly the willingness to switch chemotherapy decisions when genomic risk assessment is discordant with the physician’s original, clinically based recommendation. The primary objective of the prospective WSG-PRIMe study was to determine the impact of MammaPrint and BluePrint on systemic adjuvant chemotherapy decisions in hormone receptor (HR)-positive HER2-negative breast cancer in a representative German population. The study was also intended to probe underlying psychological factors correlated to observed levels of adherence, particularly in patients with discordance between genomic and clinical risk assessments or intrinsic subtype classification.

## Materials and methods

The prospective, observational multicenter WSG-PRIMe study was designed to quantify the impact of MammaPrint and BluePrint on adjuvant CT treatment decisions in early-stage breast cancer patients for whom MammaPrint was considered as part of their normal clinical procedure, specifically to demonstrate an overall switch percentage of at least 15% regarding chemotherapy. Female patients ≥ 18 years of age with histologically proven invasive pT1-3, pN0-1, HR-positive, HER2-negative early breast cancer were enrolled in the trial, after signing informed consent. Patients with ≥ 4 involved axillary nodes, multicentric or metastatic disease, or prior malignancies within the past 5 years were excluded.

ER/PR positivity was determined locally (IHC cutoff generally 1% positive); HER2 negativity was defined as IHC 0–1+, or FISH or other ISH non-amplified. Pathology was performed locally and included Ki-67 measurements. Clinical subtype assessment was based on the St. Gallen 2013 guidelines [8].

**Fig. 1:**
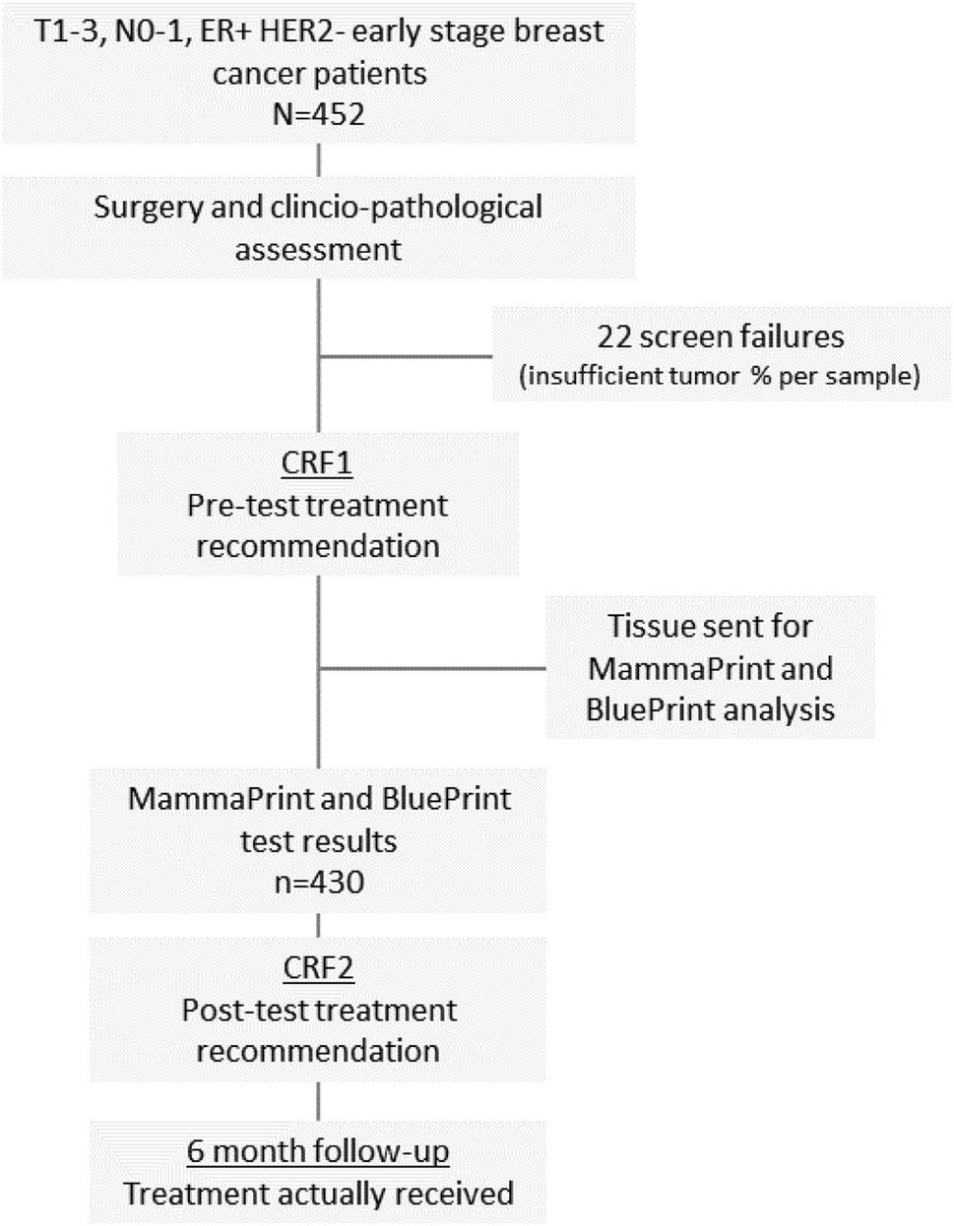
Study flowchart: tumors from 452 luminal early-stage breast cancer patients were examined using standard clinical and pathological methods. The first CRF was used to capture the relevant clinicopathological data as well as the impact of clinicopathological results on treatment recommendation prior to MammaPrint testing. Inclusion of a pre-test (post-surgery) chemotherapy recommendation by the physician was mandatory. Tumor samples were sent for genomic analysis to Agendia N.V. (Amsterdam, the Netherlands) and analyzed centrally. Once risk classification and molecular subtyping was available and the physician had discussed the result with the patient, the second CRF was completed. A track change month follow-up was conducted to determine therapy actually received

Between April 2015 and March 2016, 452 patients were enrolled in 27 centers in Germany (23), Austria (1), and Switzerland (3). Figure 1 details the study flow chart: Electronic CRFs were used to capture relevant clinicopathological data, impact of test results on treatment recommendations, and actual treatment received. Both CRFs (pre and post-test) included standard, validated decisional conflict and state-trait anxiety inventory (STAI) questionnaires and questions addressing the investigators’ confidence in treatment recommendations.

MammaPrint and Blueprint analysis was carried out centrally by Agendia N.V. (Amsterdam, the Netherlands). A minimum tumor percentage of 30% in the tissue sample was required to obtain a valid result. Once risk classification and molecular subtyping had become available and the physician had discussed the result with the patient, the second CRF was completed.

Adjuvant chemotherapy and endocrine therapy were administered according to national guidelines (for German centers: AGO 2014–2015; http://www.ago-online.de). A 6-month follow-up was conducted to determine therapy actually received (which could still differ from “post-test” recommendations).

The study was carried out under auspices of the West German Study Group (WSG, Protocol number: WSG-DI-02) and was approved by the medical ethics committee of the University of Munich (LMU, Munich, Germany) and the review boards of the individual centers.

## Statistics

The WSG-PRIMe study was powered to test the hypothesis of an overall switch proportion of at least 15% with a one-sided proportion test with alpha = 0.025. This test had at least 80% power for *N* = 430 and assumed switch proportion 20%. The key secondary objective was to assess the impact of MammaPrint on adjuvant treatment decisions separately within discordant groups (initial physician chemotherapy recommendation discordant with MammaPrint) by summary statistics.

Summary statistics with 95% confidence intervals were also computed for further secondary objectives: change in patients’ decisional conflict status and anxiety levels before and after MammaPrint results and change in investigators’ confidence in treatment recommendations before vs. after MammaPrint results (overall and stratified by risk group). Paired *t* tests were used to compute two-sided *p* values for pre vs. post-MammaPrint comparisons of continuous variables. The McNemar–Bowker test was used to compare ordinal variables pre- vs. post-MammaPrint. Continuously distributed variables in unpaired groups were compared using *t* tests.

## Results

### Patient characteristics

Within a 1-year period, 452 EBC patients were enrolled in the WSG-PRIMe study; 430 patients were evaluable, following 22 screening failures due to poor sample quality. Table 1 describes population characteristics: Median age was 58 years, with 23% (*n* = 97) < 50 years, while 70% were post-menopausal. About 28% were node positive; two-thirds were pT1.

**Table 1:** Patient and tumor characteristics

Patient characteristics (*n* = 430)	
Age in years (median)	58 (33–88)
Menopausal status	
Post-menopausal	292 (67.9%)
Pre-menopausal	127 (29.5%)
Unknown	11 (2.6%)
Tumor Characteristics	
pT1	287 (66.7%)
pT2	130 (30.2%)
pT3	13 (3.0%)
Lymph node negative	309 (71.9%)
Lymph node positive (1–3)	121 (28.1%)
Receptor status (IHC/FISH)	
ER positive	429 (99.8%)
PR positive	388 (90.2%)
HER2 negative	428 (99.5%)
HER2 unknown	2 (0.5%)

### MammaPrint risk assignment and physicians’ recommendations

**Fig. 2:**
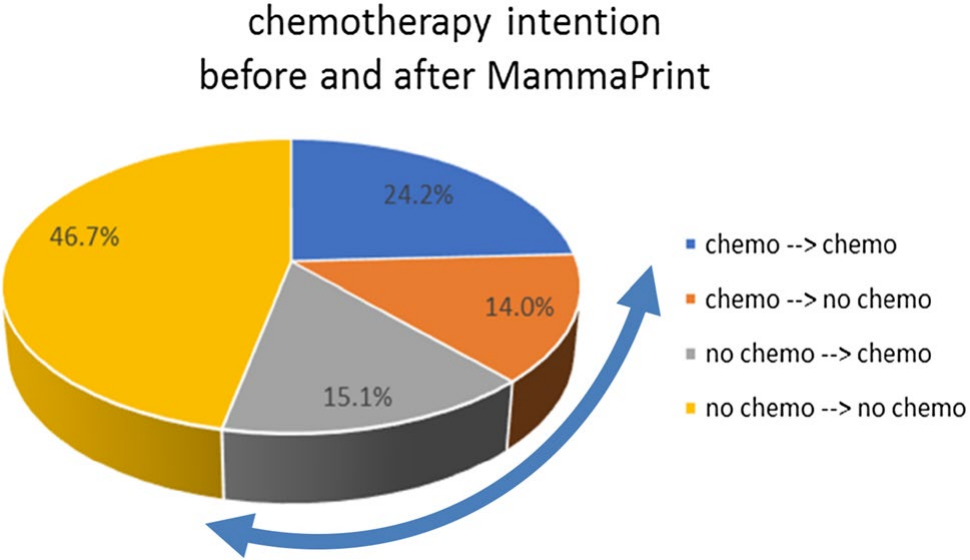
Chemotherapy recommendation before and after MammaPrint test result (arrow indicates switch proportion)

MammaPrint assigned 63.5% of patients to the low-risk category and 36.5% to the high-risk category. In almost one-third (125/430, 29.1%) of patients (95% CI 24.8–33.6%), the recommendation changed from chemotherapy to no chemotherapy or vice versa. Since the lower confidence limit of switch percentage exceeded 15%, the study achieved its primary objective, i.e., the null hypothesis was rejected (*p* < 0.001). Figure 2 illustrates CT post- vs. pre-test recommendations in the study population.

Table 2 summarizes physicians’ pre- vs. post-test recommendations, stratified by MammaPrint risk assessment. CT had been recommended to 164 patients (38.1%) pre-test. In 60/164 (36.1%) of these patients, the treatment recommendation switched to omission of CT post-test; most (59/60, 98%) of these switches occurred in MammaPrint low-risk patients. Conversely, omission of CT had been recommended to 266 patients (61.9%) pre-test; in 65/266 (24.4%) cases, recommendations switched to CT post-test; most (64/65, 98.4%) of these switches occurred in MammaPrint high-risk patients.

**Table 2:** Switch in chemotherapy (CT) decision based on MammaPrint low-risk and high-risk classification

Post-test recommendation	Pre-test recommendation							
	CT				No CT			
	CT	No CT	Total	Percentage switch CT → no CT (%)	No CT	CT	Total	Percentage switch No CT → C(%)
All patients	104	60	164	36.6	201	65	266	24.4
MammaPrint high risk	84	1	85	1.2	8	64	72	88.9
MammaPrint low risk	20	59	79	74.7	193	1	194	0.5

Pre-test recommendations for CT or no CT (based on clinicopathological results prior to MammaPrint testing) are subdivided by the subsequent post-test chemotherapy decisions. Percentage switch from CT to no CT and vice versa is displayed for total patient numbers and according to MammaPrint low-risk and high-risk results. For patients with an initial CT recommendation, 74.7% of physicians switched to CT omission following Low-Risk MammaPrint results. Conversely, 88.9% of physicians initially recommending CT omission switched to CT recommendations following High-Risk MammaPrint results. Treatment recommendations remained unchanged in almost all cases where the initial CT recommendation was concordant with genomic test results

**Fig. 3:**
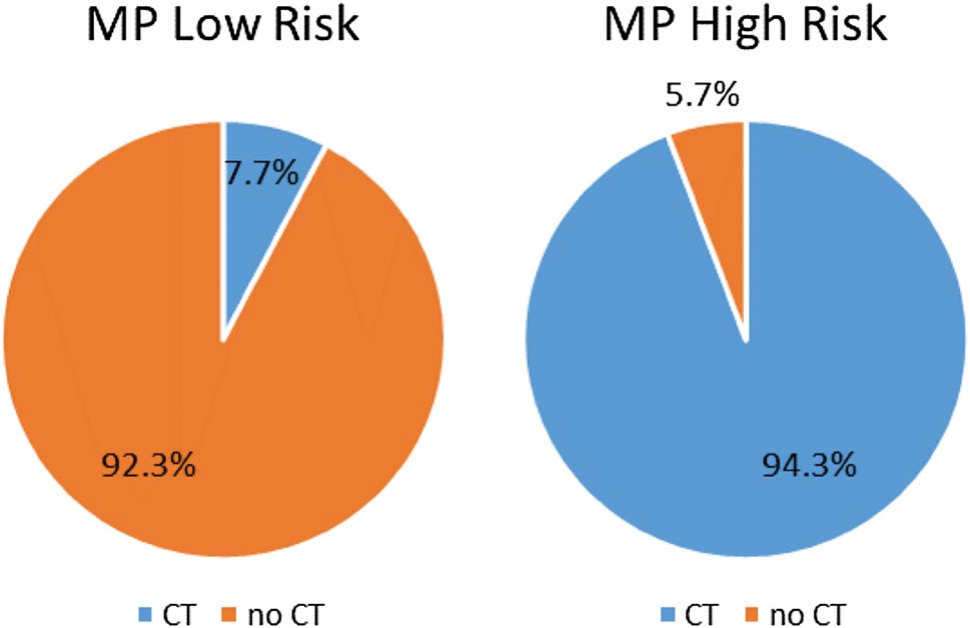
Percentage adherence to MammaPrint (MP) test result with respect to chemotherapy (CT) post-test chemotherapy recommendation

Physician adherence to MammaPrint test results was remarkably high: Overall physician adherence to MammaPrint risk assessment was 92.3% for low-risk and 94.3% for high-risk scores (Fig. 3). Three-fourths (*n* = 59/79, 74.7%) of physicians initially recommending CT switched to CT omission following low-risk MammaPrint results (72.7% in pN0, 77.1% in pN1); conversely, almost nine-tenths (*n* = 64/72, 88.9%) of physicians initially recommending CT omission switched to CT recommendations following high-risk MammaPrint results (88.1% in pN0, 92.3% in pN1). Treatment recommendations were unchanged in nearly all cases where the initial adjuvant systemic therapy recommendation was concordant with the genomic test result; 99.5% of physicians adhered to their initial recommendation of chemotherapy omission on receipt of low-Risk MammaPrint results, and 98.8% of physicians adhered to their initial chemotherapy recommendation following high-risk MammaPrint results (Table 2).

### Actual treatment vs. physicians’ recommendations

Of the 254 patients with a post-test physician recommendation to forgo chemotherapy and with known actual treatment, 252 (99.2%) actually received no chemotherapy. However, among the 160 patients with post-test chemotherapy recommendations and known actual treatment, 34 (21.3%) did not receive chemotherapy.

Remarkably, among patients whose physicians recommended chemotherapy both pre- and post-tests, 8/20 (40.0%) of patients with a MammaPrint low-risk result finally ended up forgoing chemotherapy, compared to 12/81 (14.8%) with MammaPrint high-risk (*p* = 0.01). In addition, 13/58 (22.4%) of patients whose physicians had changed from ET to chemotherapy recommendation following high-risk MammaPrint results did not receive chemotherapy.

The average age of patients forgoing chemotherapy despite high-risk MammaPrint and post-test physician’s chemotherapy recommendation was 63.8 years, compared to 57.5 years among all others (*p* < 0.001).

### Impact of BluePrint

BluePrint classifies breast tumors within four intrinsic subtypes: Luminal A, Luminal B, HER2, and Basal type. Concordance between IHC assessment and BluePrint subtyping of Luminal A or B-like tumors was seen in only 65.3% of the patients. Two clinical Luminal A-like tumors and four Luminal B-like tumors were reclassified by BluePrint to Basal type. Forty-six percent (80/173) of Luminal B-like tumors were reclassified by BluePrint to Luminal A, and 24% (62/256) of Luminal A-like tumors were assigned to Luminal B (Table 3). The overall discordance in subtype classification was 34%.

**Table 3:** Tumor reclassification by BluePrint

Clinical subtype	BluePrint/MammaPrint			
	Luminal A	Luminal B	Basal	Total
Luminal A-like	192 (75%)	62 (24%)	2 (1%)	256
Luminal B-like	80 (46%)	89 (51%)	4 (2%)	173
HER2	0	1 (100%)	0	1
Total	272	152	6	430

CT recommendations were strongly associated with molecular subtype: 143 of the 152 molecular Luminal B patients (94.1%) received a CT recommendation, while 251 of the 272 molecular Luminal A subjects (92.3%) received a recommendation to omit CT (Table 4).

**Table 4:** Adherence to CT decision based on BluePrint/MammaPrint classification

Post-test recommendation	BluePrint/MammaPrint			
	Luminal A	Luminal B	Basal	Total
CT	21 (7.7%)	143 (94.1%)	5 (83.3%)	169
No CT	251 (92.3%)	9 (5.9%)	1 (16.7%)	261
Total	272	152	6	430

### Physicians’ confidence and perceptions

Table 5 summarizes physician’s confidence in treatment recommendation pre- vs. post-test result by MammaPrint risk category and BluePrint classification. Increases in confidence were prominent in both risk groups (low-risk, *p* < 0.001; high-risk, *p* = 0.001). Improvements in confidence occurred in 32.9% of cases overall (29.9% in high-risk and 34.6% in low-risk cases), compared to 57.1% unchanged (60.5% in high-risk and 55.1% in low-risk cases) and 10.0% lower confidence (9.6% in high-risk and 10.3% in low-risk patients, respectively). The percentage of physicians with complete or high confidence in their treatment recommendations increased overall from 68.6% pre-test to 85.1% post-test; the improvement was 65.0–83.4% in MammaPrint high-risk and 70.7–84.9% in MammaPrint low-risk cases.

**Table 5:** Physician’s confidence in treatment recommendation pre- and post-test result by risk group

Pre vs. post-test physician confidence	Physicians’ pre-test confidence in treatment recommendation					
	Complete	High	Intermediate	Low	Total	
Physicians’ post-test confidence in treatment recommendation						
MP high risk						
Complete	3	8	2	0	13	
High	6	77	32	3	118	
Intermediate	0	8	15	2	25	
Low	0	0	1	0	1	
Total	9	93	50	5	157	
MP low risk						
Complete	6	36	8	3	53	
High	10	127	39	5	181	
Intermediate	2	11	13	3	29	
Low	0	1	4	4	9	
Missing	0	0	1	0	1	
Total	18	175	65	15	273	
Total						
Complete	9	44	10	3	66	84.90%
High	16	204	71	8	299	
Intermediate	2	19	28	5	54	
Low	0	1	5	4	10	
Missing	0	0	1	0	1	
	27	268	115	20	430	
	68.60%					

Overall “complete” or “high” confidence rates increased from 68.6% pre-test to 84.9% post-test

### Patients’ decisional conflict and state-trait anxiety inventory

Patients’ decisional conflict scores (on a scale of 100) improved significantly overall and within risk groups following MammaPrint. The mean changes were − 12.78 points [95% CI − 14.83 to − 10.73] overall; − 8.00 [95% CI − 11.31 to − 4.67] in high-risk; − 15.51 [95% CI − 18.07 to − 12.95] in low-risk patients (*p* < 0.001 for all comparisons).

**Fig. 4:**
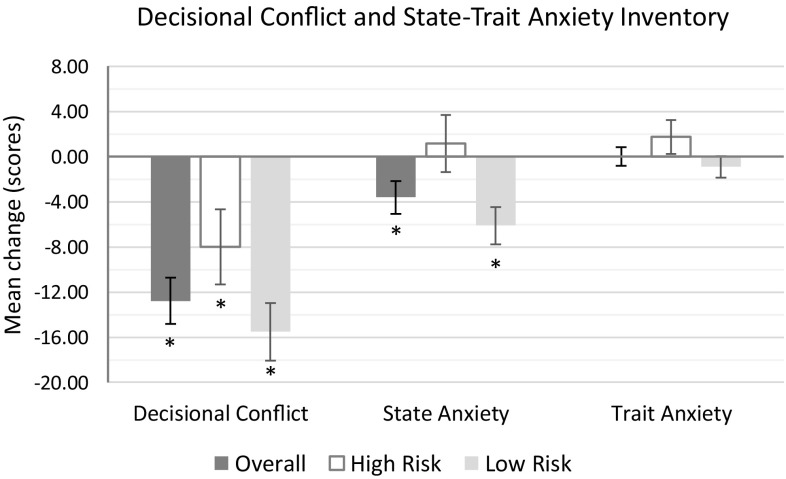
Mean change in patient decisional conflict, state anxiety (current), and trait anxiety (long-term) scores. Negative changes represent improvements (decreases in conflict/anxiety). Error bars represent 95% confidence intervals, *p < 0.001

Overall, patients’ State Anxiety scores improved (decreased) significantly (Table 6; Fig. 4). The mean change was − 3.61 points [− 5.06 to − 2.16] (*p* < 0.001). However, there was a remarkable difference between high- and low-risk subsets: In high-risk patients, there was a slight (non-significant) mean increase, i.e., the change was + 1.17 points [− 1.36 to 3.69]; in low-risk patients, there was a significant (*p* < 0.001) decrease, i.e., the mean change was − 6.11 points [− 7.78 to − 4.44].

**Table 6:** Summary of patient decisional conflict score, State Anxiety, and Trait anxiety scores

	Mean change	95% confidence interval		*p* value
Patients’ decisional conflict score				
Overall	− 12.78	− 14.83	− 10.73	< 0.001
High risk	− 8.00	− 11.31	− 4.67	< 0.001
Low risk	− 15.51	− 18.07	− 12.95	< 0.001
Patients’ state anxiety score				
Overall	− 3.61	− 5.06	− 2.16	< 0.001
High risk	+ 1.17	− 1.36	+ 3.69	NS
Low risk	− 6.11	− 7.78	− 4.44	< 0.001
Patients’ trait anxiety score				
Overall	+ 0.008	− 0.81	+ 0.82	NS
High risk	+ 1.76	+ 0.26	+ 3.26	0.02
Low risk	− 0.90	− 1.86	+ 0.04	0.06

*NS* non-significant

Overall, patients’ Trait Anxiety scores remained virtually unchanged (increase of 0.008, [− 0.81 to 0.82]). In high-risk patients, there was a modest but significant (*p* = 0.02) average increase of 1.76 [0.26 to 3.26] in Trait Anxiety scores. In low-risk patients, there was a slight (*p* = 0.06, borderline significant) decrease in Trait Anxiety: the mean change was − 0.90 points [− 1.86 to + 0.04].

MammaPrint test risk level and post-test chemotherapy recommendations were both strongly associated with final (post-test) decisional conflict, state anxiety, and (to a lesser extent) Trait Anxiety scores (Table 7, *p* < 0.001 in all comparisons). Mean post-test decisional conflict scores were 6.9 points higher (18.7 vs. 11.8) in MammaPrint high-risk than in MammaPrint low-risk patients; mean post-test state anxiety scores were 11.4 points higher (45.5 vs. 34.1); and mean post-test trait anxiety scores were 4.7 points higher (37.8 vs. 33.1). In patients with recommended chemotherapy, mean post-test decisional conflict scores were 6.7 points higher (18.4 vs. 11.7) than those with recommended chemotherapy omission; mean post-test state anxiety scores were 10.5 points higher (44.6 vs. 34.1); and mean post-test trait anxiety scores were 5.0 points higher (37.8 vs. 32.8).

**Table 7:** Summary of decisional conflict score, State Anxiety, and Trait anxiety scores post chemotherapy decision

	High risk	Low risk	*p* value
High vs. low risk (mean post-test scores)			
Decisional conflict	18.7	11.8	< 0.001
State anxiety	45.5	34.1	< 0.001
Trait anxiety	37.8	33.1	< 0.001
Chemotherapy decision (mean post-test scores)			
Decisional conflict	18.4	11.7	< 0.001
State anxiety	44.6	34.1	< 0.001
Trait anxiety	37.8	32.8	< 0.001

Within the high-risk MammaPrint group, discordance between the initial chemotherapy recommendation and the MammaPrint test result did not have a significant impact on Decisional Conflict scores or either anxiety score. However, within the low-risk MammaPrint group, the concordant subgroup (i.e., an initial ET recommendation and low-risk MammaPrint result) had lower Decisional Conflict scores, (− 4.6 points, *p* = 0.026) than the discordant subgroup; they also had lower State Anxiety (− 3.2 points, borderline significant, *p* = 0.06) and Trait Anxiety (− 3.7 points, *p* = 0.017).

## Discussion

The WSG-PRIMe study demonstrates that the use of the gene expression profiles, MammaPrint and BluePrint, has a strong impact on adjuvant therapy recommendations. Physicians changed their final recommendation for systemic treatment in 29.1% of cases following MammaPrint testing. The most striking influences were observed in MammaPrint groups that were “discordant” with the physician’s initial chemotherapy recommendation: about three-fourths of physicians initially recommending CT switched to CT omission following low-risk MammaPrint results, and almost nine-tenths of physicians initially recommending CT omission switched to CT recommendations following high-risk MammaPrint results. Switches were very rare in “concordant” situations, so that the switches are almost certainly attributable to the impact of the genomic testing results, and the resulting overall physician adherence to MammaPrint risk assessment was very high (92.3% for low-risk and 94.3% for high-risk categories).

Moreover, concerning chemotherapy actually administered, the impact of MammaPrint risk assessment persisted (or even intensified), with 40% of patients whose physicians had recommended chemotherapy both pre- and post-tests despite a MammaPrint low-risk result finally forgoing chemotherapy, compared to 15% with the same recommendation sequence and MammaPrint high risk.

Impact studies in other European countries have generally shown similar switch rates: Belgium (24%), Italy (28%), Spain (35%), and Austria (19%) [16, 17], while higher switch rates were observed in South Africa (52%) [18] and the US (33.6%) [19]. Taken together, these studies appear to confirm an international trend in physicians toward increased confidence in the applicability of genomic testing to inform breast cancer treatment options.

In a recent Dutch impact trial, Kuijer et al. [20] reported a 51% switch rate in CT treatment recommendation—following MammaPrint risk classification—among those physicians who had a definite pre-test recommendation (“sure group”). However, this rate is not directly comparable to the 29% switch rate observed here: first, the pre-test recommendation in favor of chemotherapy in the “sure” pre-test group of Kujer et al. was 72%, or almost double the rate (about 38%) in the present WSG-PRIMe study. Moreover, because the Kuijer et al. trial design included an “unsure” pre-test group, the switch rate in the “sure” pre-test group is unlikely to be representative for the rate that would have been observed with the WSG-PRIMe study design (mandatory pre-test chemotherapy preference). There was a net *increase* of about 1.2% in chemotherapy recommendation post-test vs. pre-test in the WSG-PRIMe study—far removed from the 34% *decrease* that can be calculated among “sure” pre-test patients from Table 2 of Kujer et al. [20]. However, comparisons of total chemotherapy recommendation changes across trials are complicated by (1) differing study designs (as above); (2) dependence on methodology used by physicians to determine pre-test risk; and (3) sensitivity to clinicopathological risk distributions of trial populations. (Switch rates in discordant groups (low-clinical/high-genomic risk and vice versa), as estimates of conditional probabilities, may be less sensitive to some of these issues.) Post-test adherence to genomic test results was 96% in the Kuijer et al. study [20], quite comparable to the 92.3% adherence for low-risk and 94.3% for high-risk MammaPrint categories, as seen in the WSG-PRIMe study. The overall high adherence to genetically determined risk assessment (in both trials) represents a key prerequisite for achieving a more targeted approach to disease management in patients with early-stage breast cancer.

Decisional conflict arises when a person is confronted with high-stakes choices (as in the chemotherapy decision in ER-positive, HER2-negative early breast cancer); it can be mitigated by decision supporting interventions [21, 22]. Following MP risk assessment and the ensuing discussion with the physician, patients’ decisional conflict scores improved (decreased) significantly overall and separately within both the MP low-risk and (remarkably) the MP high-risk groups. The results are consistent with the interpretation that use of (MP-based) risk assessment helped to reduce the uncertainty associated with benefits and risks, leading to patients’ perception of a more informed, objectively justified, and empowered chemotherapy decision.

State Anxiety scores capture the current state of anxiety, e.g., at time of a stressful event or perceived threat. Overall, State Anxiety scores improved (decreased) significantly following MP assessment. However, most of this improvement can be attributed to the MP low-risk patients, who had a substantial and significant decrease (about 6 points on the scale from 20 to 80), whereas in high-risk patients there was a slight (non-significant) mean increase in anxiety.

As seen above, State Anxiety (and Decisional Conflict) were higher (more severe) in patients receiving a post-test chemotherapy recommendation. Since final chemotherapy recommendation was strongly associated with MammaPrint risk (*p* < 0.001), patients’ increased anxiety could be attributable to chemotherapy per se or the threat associated with a more severe form of breast cancer or both. Indeed, among patients with a final chemotherapy recommendation, the post-test State Anxiety scores in high-risk cases exceeded those in low-risk cases by almost 10 points (45.9 vs. 36.2). This difference was significant (*p* = 0.009), although (due to high adherence) only about 13% of those with post-test chemotherapy recommendations (and complete state anxiety score data) had low-risk MammaPrint tests. Among post-test high-risk patients with complete State Anxiety data, anxiety scores were about seven points lower among the few (6) patients without a post-test chemotherapy recommendation (not significant). Within the low-risk MammaPrint group, the concordant subgroup (i.e., those with initial ET recommendation and low-risk MammaPrint result) had less severe Decisional Conflict, State Anxiety, and Trait Anxiety. Increased anxiety thus appears to be independently attributable to both chemotherapy and the threat associated with an MP high-risk result, i.e., the perception of a more severe form of breast cancer.

Trait Anxiety scores are designed to quantify long-term traits, e.g., general states of calmness, confidence, and security. While average Trait Anxiety Scores remained unchanged overall, the risk groups responded differently: in high-risk patients, there was a modest but significant average post-test increase in Trait Anxiety scores, whereas in low-risk patients, there was a slight decrease.

MINDACT and other studies suggest that a substantial group of patients with clinical high-risk profile but low genomic risk may be candidates for avoiding chemotherapy, with the advantage of improved quality of life and a lower incidence of adverse effects. In the past, guidelines based on clinicopathological risk factors have often erred in the direction of overtreatment. Following the publication of the MINDACT trial [15], the AGO guidelines were updated, giving MammaPrint a level 1A recommendation to justify withholding potentially unnecessary chemotherapy. The German S3 guideline (2017) supports the use of gene expression assays when clinicopathological criteria do not indicate a preferred treatment. However, the true merit of genomic assessment lies in the increased probability that breast cancer patients can receive the treatment that best matches their specific tumor biology.

Recently, molecular subtyping has increasingly been applied to support treatment selection in breast cancer. However the identification of proteins by IHC/FISH does not necessarily indicate protein functionality and hence tumor behavior. BluePrint not only measures whether these commonly used biomarker proteins are expressed, but also incorporates additional information on expression patterns in the underlying biological pathways regulated by these proteins (e.g., estrogen receptor (ER) and downstream ER targets). These features enable better prediction of tumor behavior and potential efficacy of targeted therapy [23]. In neoadjuvant trials, positive pathological complete response (pCR) is increasingly accepted as a surrogate for efficacy regarding survival, especially if substantial impact on pCR can be demonstrated. BluePrint reclassification of patients leads to a more striking differentiation of pCR rates by subgroup: lower pCR in BluePrint-luminal patients compared with IHC/FISH determination, with more responsive patients reassigned to HER2 and Basal categories [24–26]. BluePrint molecular subtyping reclassified 9–22% of tumors compared with IHC [26, 27]. The 34% discordance between clinical and molecular subtypes found here underlines the potential impact of genomic testing in EBC.

Ten-year follow-up data in patients receiving systemic adjuvant therapy [28] and 5-year outcome data in the neoadjuvant setting [7] have shown that BluePrint classification is more strongly associated with CT response than IHC/FISH subtyping. In the MINDACT study, 16% of the patients were re-stratified to the low-risk luminal A group by BluePrint and had 95% 5y-DMFS [15, 29]. These studies demonstrate the prognostic power of MammaPrint and importance of classification of tumors by molecular subtype according to BluePrint.

In the present prospective, multicenter decision impact study in ER-positive, HER2-negative EBC, MammaPrint, and BluePrint had a substantial impact on physicians’ treatment recommendations. The very high adherence in the WSG-PRIMe Study, especially the high switch rates in discordant groups (low-clinical/high-genomic risk and vice versa)—supported by decisional conflict and confidence results of physicians—suggests that most physicians were convinced that patients with MammaPrint high-risk tumors (most of which were classified by BluePrint as Luminal B or occasionally Basal) would benefit from chemotherapy, and that MammaPrint low-risk patients (almost all Luminal A by BluePrint) could profit from omission of chemotherapy. The observed 29% change in treatment advice and very high (93%) adherence to the genomic test results suggest that physicians recognize the importance of a personalized approach that integrates genomic, clinical, and pathological factors to guide patient treatment. The results also suggest that patients in this intrinsically high-stakes situation (chemotherapy decision) feel empowered by the support to the decision-making process afforded by MP assessment, as evidenced by reduction of anxiety even in high-risk patients.

More generally, improved, genomically determined individualization of treatment regimens could lead to a decreased risk of over- *or* undertreatment of patients. The overall high adherence to genomically determined risk assessment represents a key prerequisite for achieving a more targeted and cost-effective approach to disease management in early-stage breast cancer.
